# 1-Methyl-1,3-diazinan-2-one

**DOI:** 10.1107/S1600536812016522

**Published:** 2012-04-21

**Authors:** Ioannis Tiritiris, Willi Kantlehner

**Affiliations:** a Institut für Organische Chemie, Universität Stuttgart, Pfaffenwaldring 55, 70569 Stuttgart, Germany; b Fakultät Chemie/Organische Chemie, Hochschule Aalen, Beethovenstrasse 1, D-73430 Aalen, Germany

## Abstract

In the crystal structure of the title compound, C_5_H_10_N_2_O, mol­ecules are connected *via* pairs of strong N—H⋯O hydrogen bonds into centrosymmetric dimers, which are stacked along the *a* axis. The molecule is not planar, the dihedral angle between the N/C/N and C/C/C planes being 42.1(1)°.

## Related literature

For substitution of hexa­methyl­phospho­ramide (HMPT) by the cyclic urea 1,3-dimethyl-3,4,5,6-tetra­hydro­pyrimidin-2-one (DMPU), see: Mukhopadhyay & Seebach (1982 ▶). For the crystal structure of 3,4,5,6-tetra­hydro­pyrimidin-2-one, see: Rizal *et al.* (2008 ▶) and of 1-methyl-imidazolidin-2-one, see: Caudle *et al.* (2005 ▶).
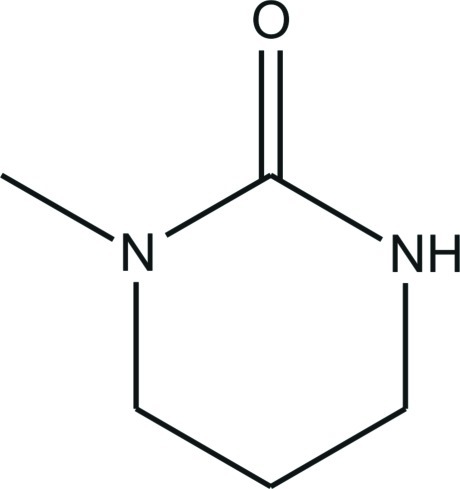


## Experimental

### 

#### Crystal data

C_5_H_10_N_2_O
*M* *_r_* = 114.15
Orthorhombic, 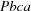

*a* = 5.8479 (2) Å
*b* = 13.3438 (6) Å
*c* = 15.0883 (8) Å
*V* = 1177.39 (9) Å^3^
*Z* = 8
Mo *K*α radiation
μ = 0.09 mm^−1^
*T* = 100 K
0.19 × 0.15 × 0.11 mm

#### Data collection

Bruker–Nonius KappaCCD diffractometer
2628 measured reflections
1434 independent reflections
1190 reflections with *I* > 2σ(*I*)
*R*_int_ = 0.024

#### Refinement

*R*[*F* ^2^ > 2σ(*F* ^2^)] = 0.037
*wR*(*F* ^2^) = 0.092
*S* = 1.03
1434 reflections
79 parameters
H atoms treated by a mixture of independent and constrained refinement
Δρ_max_ = 0.27 e Å^−3^
Δρ_min_ = −0.18 e Å^−3^

### 

Data collection: *COLLECT* (Hooft, 2004 ▶); cell refinement: *SCALEPACK* (Otwinowski & Minor, 1997 ▶); data reduction: *SCALEPACK*; program(s) used to solve structure: *SHELXS97* (Sheldrick, 2008 ▶); program(s) used to refine structure: *SHELXL97* (Sheldrick, 2008 ▶); molecular graphics: *DIAMOND* (Brandenburg & Putz, 2005 ▶); software used to prepare material for publication: *SHELXL97*.

## Supplementary Material

Crystal structure: contains datablock(s) I, global. DOI: 10.1107/S1600536812016522/fk2059sup1.cif

Structure factors: contains datablock(s) I. DOI: 10.1107/S1600536812016522/fk2059Isup2.hkl

Supplementary material file. DOI: 10.1107/S1600536812016522/fk2059Isup3.cml

Additional supplementary materials:  crystallographic information; 3D view; checkCIF report

## Figures and Tables

**Table 1:** Hydrogen-bond geometry (Å, °)

*D*—H⋯*A*	*D*—H	H⋯*A*	*D*⋯*A*	*D*—H⋯*A*
N1—H1⋯O1^i^	0.88 (2)	2.00 (2)	2.875 (1)	177 (2)

Symmetry code: (i) 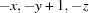.

**Fig. 1.**
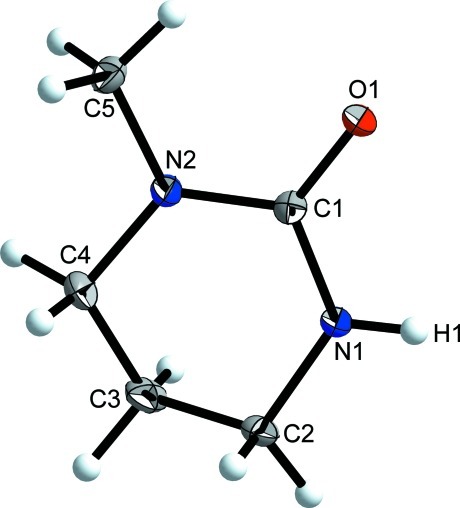
The molecular structure of the title molecule. Anisotropic displacement ellipsoids are shown at the 50% probability level.

**Fig. 2.**
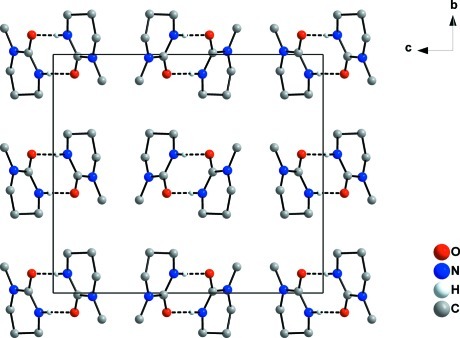
Packing diagram of the title compound, bc-view. The N–H···O hydrogen bonds are indicated by dashed lines.

## supplementary crystallographic information

### Comment

1,3-Dimethyl-3,4,5,6-tetrahydropyrimidin-2-one (DMPU), a liquid at room temperature, is often used in organic synthesis as a polar aprotic solvent, replacing the carcinogenic hexamethylphosphoramide (HMPT) (Mukhopadhyay & Seebach, 1982). In contast, 3,4,5,6-tetrahydropyrimidin-2-one is a solid with a melting point of 263–267 °C and its ordered crystal structure was quite recently determined (Rizal *et al.*, 2008). The crystal structure of the missing link 1-methyl-3,4,5,6-tetrahydropyrimidin-2-one (I) was previously unknown. Prominent bond parameters for the title molecule are: C1–O1 = 1.248 (1) Å, N1–C1 = 1.357 (1) Å and N2–C1 = 1.362 (1) Å. The bond length between N2 and the terminal *C*-methyl group (C5) measures 1.453 (1) Å. The C–N2–C angles are: 123.54 (9)° (C4–N2–C1), 120.32 (8)° (C1–N2–C5) and 115.18 (9)° (C5–N2–C4), which indicates a trigonal-planar surrounding of the nitrogen centre by the C atoms. These data are in good agreement with those of the five membered heterocycle 1-methyl-imidazolidin-2-one (Caudle *et al.*, 2005). In contrast to the aforementioned compound, the six membered heterocycle in (I) is non–planar (Fig. 1). The carbon atom C3 is not in the ring plane, the angle between the planes N1/C1/N2 and C2/C3/C4 is 42.1 (1)°. In the packing, each two molecules are linked by strong N–H···O hydrogen bonds, forming centrosymmetric dimers, which are stacked along the *a* axis (Fig.2). The H···O distance is 2.00 (2) Å, with a nearly linear N–H···O angle of 177 (2)° (Tab.1).

### Experimental

The title compound was obtained as a byproduct by reaction of 1-Methyl-2-dimethylamino-1,4,5,6-tetrahydropyrimidinium-chloride with excess aqueous sodium hydroxide at room temperature. After distillation of the crude product *in vacuo*, a colourless liquid was obtained. The compound crystallized spontaneously upon standing at room temperature after several days, forming colourless single crystals.

### Refinement

The N-bound H atom was located in a difference Fourier map and was refined freely. The hydrogen atoms of the methyl group were allowed to rotate with a fixed angle around the C–N bond to best fit the experimental electron density, with *U*(H) set to 1.5 *U*_eq_(C) and d(C—H) = 0.98 Å. The remaining H atoms were placed in calculated positions with d(C—H) = 0.99 Å and were included in the refinement in the riding model approximation, with *U*(H) set to 1.2 *U*_eq_(C).

### Crystal data

**Table:**

C_5_H_10_N_2_O	*F*(000) = 496
*M**_r_* = 114.15	*D*_x_ = 1.288 Mg m^−^^3^
Orthorhombic, *P**b**c**a*	Mo *K*α radiation, λ = 0.71073 Å
Hall symbol: -P 2ac 2ab	Cell parameters from 1677 reflections
*a* = 5.8479 (2) Å	θ = 0.4–28.3°
*b* = 13.3438 (6) Å	µ = 0.09 mm^−^^1^
*c* = 15.0883 (8) Å	*T* = 100 K
*V* = 1177.39 (9) Å^3^	Lath-shaped, colourless
*Z* = 8	0.19 × 0.15 × 0.11 mm

### Data collection

**Table:**

Bruker–Nonius KappaCCD diffractometer	1190 reflections with *I* > 2σ(*I*)
Radiation source: sealed tube	*R*_int_ = 0.024
Graphite monochromator	θ_max_ = 28.2°, θ_min_ = 2.7°
φ scans, and ω scans	*h* = −7→7
2628 measured reflections	*k* = −17→17
1434 independent reflections	*l* = −19→19

### Refinement

**Table:**

Refinement on *F*^2^	Secondary atom site location: difference Fourier map
Least-squares matrix: full	Hydrogen site location: difference Fourier map
*R*[*F*^2^ > 2σ(*F*^2^)] = 0.037	H atoms treated by a mixture of independent and constrained refinement
*wR*(*F*^2^) = 0.092	*w* = 1/[σ^2^(*F*_o_^2^) + (0.0424*P*)^2^ + 0.4078*P*] where *P* = (*F*_o_^2^ + 2*F*_c_^2^)/3
*S* = 1.03	(Δ/σ)_max_ < 0.001
1434 reflections	Δρ_max_ = 0.27 e Å^−^^3^
79 parameters	Δρ_min_ = −0.18 e Å^−^^3^
0 restraints	Extinction correction: *SHELXL97* (Sheldrick, 2008), Fc^*^=kFc[1+0.001xFc^2^λ^3^/sin(2θ)]^-1/4^
Primary atom site location: structure-invariant direct methods	Extinction coefficient: 0.023 (3)

### Special details

**Table:**

Geometry. All e.s.d.'s (except the e.s.d. in the dihedral angle between two l.s. planes) are estimated using the full covariance matrix. The cell e.s.d.'s are taken into account individually in the estimation of e.s.d.'s in distances, angles and torsion angles; correlations between e.s.d.'s in cell parameters are only used when they are defined by crystal symmetry. An approximate (isotropic) treatment of cell e.s.d.'s is used for estimating e.s.d.'s involving l.s. planes.
Refinement. Refinement of *F*^2^ against ALL reflections. The weighted *R*-factor *wR* and goodness of fit *S* are based on *F*^2^, conventional *R*-factors *R* are based on *F*, with *F* set to zero for negative *F*^2^. The threshold expression of *F*^2^ > σ(*F*^2^) is used only for calculating *R*-factors(gt) *etc*. and is not relevant to the choice of reflections for refinement. *R*-factors based on *F*^2^ are statistically about twice as large as those based on *F*, and *R*- factors based on ALL data will be even larger.

### Fractional atomic coordinates and isotropic or equivalent isotropic displacement parameters (Å^2^)

**Table:**

	*x*	*y*	*z*	*U*_iso_*/*U*_eq_	
O1	0.13599 (13)	0.58154 (5)	0.07922 (5)	0.0178 (2)	
N1	0.21849 (16)	0.41809 (6)	0.05287 (6)	0.0162 (2)	
H1	0.108 (3)	0.4198 (11)	0.0134 (10)	0.032 (4)*	
N2	0.44922 (16)	0.51361 (6)	0.14565 (6)	0.0147 (2)	
C1	0.26381 (18)	0.50789 (7)	0.09125 (6)	0.0127 (2)	
C2	0.3761 (2)	0.33384 (7)	0.05379 (7)	0.0170 (2)	
H2A	0.4901	0.3415	0.0058	0.020*	
H2B	0.2913	0.2706	0.0439	0.020*	
C3	0.4956 (2)	0.33051 (8)	0.14262 (7)	0.0203 (3)	
H3A	0.6120	0.2766	0.1427	0.024*	
H3B	0.3834	0.3162	0.1901	0.024*	
C4	0.6091 (2)	0.43089 (8)	0.15938 (8)	0.0209 (3)	
H4A	0.6673	0.4330	0.2210	0.025*	
H4B	0.7411	0.4387	0.1189	0.025*	
C5	0.5230 (2)	0.60977 (8)	0.18066 (7)	0.0182 (3)	
H5A	0.4106	0.6613	0.1650	0.027*	
H5B	0.6719	0.6274	0.1553	0.027*	
H5C	0.5362	0.6055	0.2453	0.027*	

### Atomic displacement parameters (Å^2^)

**Table:**

	*U*^11^	*U*^22^	*U*^33^	*U*^12^	*U*^13^	*U*^23^
O1	0.0179 (4)	0.0130 (4)	0.0226 (4)	0.0039 (3)	−0.0052 (3)	−0.0015 (3)
N1	0.0153 (5)	0.0118 (4)	0.0213 (5)	0.0022 (4)	−0.0052 (4)	−0.0026 (3)
N2	0.0148 (5)	0.0120 (4)	0.0173 (4)	0.0003 (4)	−0.0034 (3)	0.0002 (3)
C1	0.0131 (5)	0.0122 (4)	0.0128 (4)	−0.0013 (4)	0.0008 (4)	0.0015 (4)
C2	0.0195 (5)	0.0118 (5)	0.0196 (5)	0.0036 (4)	−0.0003 (4)	−0.0011 (4)
C3	0.0260 (6)	0.0142 (5)	0.0206 (5)	0.0071 (5)	−0.0023 (5)	0.0023 (4)
C4	0.0193 (6)	0.0195 (5)	0.0241 (6)	0.0063 (5)	−0.0066 (5)	−0.0012 (4)
C5	0.0196 (5)	0.0160 (5)	0.0190 (5)	−0.0038 (4)	−0.0034 (4)	−0.0010 (4)

### Geometric parameters (Å, º)

**Table:**

O1—C1	1.2480 (13)	C2—H2B	0.9900
N1—C1	1.3570 (13)	C3—C4	1.5162 (16)
N1—C2	1.4537 (13)	C3—H3A	0.9900
N1—H1	0.881 (18)	C3—H3B	0.9900
N2—C1	1.3620 (14)	C4—H4A	0.9900
N2—C5	1.4532 (13)	C4—H4B	0.9900
N2—C4	1.4614 (14)	C5—H5A	0.9800
C2—C3	1.5123 (15)	C5—H5B	0.9800
C2—H2A	0.9900	C5—H5C	0.9800
			
C1—N1—C2	123.71 (9)	C4—C3—H3A	109.9
C1—N1—H1	114.2 (9)	C2—C3—H3B	109.9
C2—N1—H1	119.6 (10)	C4—C3—H3B	109.9
C1—N2—C5	120.32 (8)	H3A—C3—H3B	108.3
C1—N2—C4	123.54 (9)	N2—C4—C3	111.31 (9)
C5—N2—C4	115.18 (9)	N2—C4—H4A	109.4
O1—C1—N1	121.08 (10)	C3—C4—H4A	109.4
O1—C1—N2	121.36 (9)	N2—C4—H4B	109.4
N1—C1—N2	117.52 (9)	C3—C4—H4B	109.4
N1—C2—C3	108.92 (8)	H4A—C4—H4B	108.0
N1—C2—H2A	109.9	N2—C5—H5A	109.5
C3—C2—H2A	109.9	N2—C5—H5B	109.5
N1—C2—H2B	109.9	H5A—C5—H5B	109.5
C3—C2—H2B	109.9	N2—C5—H5C	109.5
H2A—C2—H2B	108.3	H5A—C5—H5C	109.5
C2—C3—C4	108.92 (8)	H5B—C5—H5C	109.5
C2—C3—H3A	109.9		
			
C2—N1—C1—O1	170.94 (10)	C1—N1—C2—C3	37.71 (14)
C2—N1—C1—N2	−11.24 (15)	N1—C2—C3—C4	−55.31 (12)
C5—N2—C1—O1	−9.34 (16)	C1—N2—C4—C3	−25.76 (14)
C4—N2—C1—O1	−177.57 (10)	C5—N2—C4—C3	165.46 (9)
C5—N2—C1—N1	172.84 (9)	C2—C3—C4—N2	50.42 (12)
C4—N2—C1—N1	4.62 (15)		

### Hydrogen-bond geometry (Å, º)

**Table:**

*D*—H···*A*	*D*—H	H···*A*	*D*···*A*	*D*—H···*A*
N1—H1···O1^i^	0.88 (2)	2.00 (2)	2.875 (1)	177 (2)

Symmetry code: (i) −*x*, −*y*+1, −*z*.
